# Genetic diversity of *Mycobacterium avium* subspecies *paratuberculosis* and the influence of strain type on infection and pathogenesis: a review

**DOI:** 10.1186/s13567-015-0203-2

**Published:** 2015-06-19

**Authors:** Karen Stevenson

**Affiliations:** Moredun Research Institute, Pentlands Science Park, Bush Loan, Penicuik, EH26 0PZ Scotland UK

## Abstract

*Mycobacterium avium* subspecies *paratuberculosis* (*Map*) is an important pathogen that causes a chronic, progressive granulomatous enteritis known as Johne’s disease or paratuberculosis. The disease is endemic in many parts of the world and responsible for considerable losses to the livestock and associated industries. Diagnosis and control are problematic, due mostly to the long incubation period of the disease when infected animals show no clinical signs and are difficult to detect, and the ability of the organism to survive and persist in the environment. The existence of phenotypically distinct strains of *Map* has been known since the 1930s but the genetic differentiation of *Map* strain types has been challenging and only recent technologies have proven sufficiently discriminative for strain comparisons, tracing the sources of infection and epidemiological studies. It is important to understand the differences that exist between *Map* strains and how they influence both development and transmission of disease. This information is required to develop improved diagnostics and effective vaccines for controlling Johne’s disease. Here I review the current classification of *Map* strain types, the sources of the genetic variability within strains, growth characteristics and epidemiological traits associated with strain type and the influence of strain type on infection and pathogenicity.

## Table of contents

1. Introduction
2. Strain types of *Mycobacterium avium* subspecies *paratuberculosis*
3. Source of genetic variability
  3.1 Large sequence polymorphisms
    3.1.1 Genomic insertions
    3.1.2 Genomic deletions
    3.1.3 Genomic inversions
    3.1.4 Genomic duplications
  3.2 Insertion sequences
  3.3 Repeat sequences
  3.4 Single nucleotide polymorphisms
  3.5 Genome stability and mutation rate
4. Genetic variability and growth characteristics
5. Genetic variability and epidemiologic traits
6. Genetic variability and virulence
7. Genetic variability, infection and pathogenesis
8. Conclusions
9. Competing interests
10. Acknowledgements
11. References

## 1. Introduction

*Mycobacterium avium* subspecies *paratuberculosis* (*Map*) is an important pathogen that causes a chronic, progressive granulomatous enteritis known as Johne’s disease or paratuberculosis. The disease is endemic in many parts of the world and responsible for considerable losses to the livestock and associated industries. Diagnosis and control are problematic, due mostly to the long incubation period of the disease when infected animals show no clinical signs and are difficult to detect, and the ability of the organism to survive and persist in the environment.

*Map* has been isolated from a diverse range of both ruminant and non-ruminant hosts [1–3] but causes clinical disease only in ruminants, camelids [4,5], rabbits [6] and hares [7]. *Map* can infect humans and has been associated with Crohn’s disease, although there is no definitive evidence and it remains a highly controversial issue [8].

The existence of phenotypically distinct strains of *Map* has been known for more than eight decades but the genetic differentiation of *Map* strain types has been challenging and only recent technologies have proven sufficiently discriminative for strain comparisons, tracing the sources of infection and epidemiological studies. The history of *Map* typing and the techniques employed has been reviewed elsewhere and will not be covered in this review [9,10]. It is important to understand the differences that exist between *Map* strains and how they influence both development and transmission of disease. This information is required to develop improved diagnostics and effective vaccines for controlling Johne’s disease. Here I review the current classification of *Map* strain types, the sources of the genetic variability within strains, growth characteristics and epidemiological traits associated with strain type and the influence of strain type on infection and pathogenicity.

## 2. Strain types of *Mycobacterium avium* subspecies *paratuberculosis*

**Figure 1:**
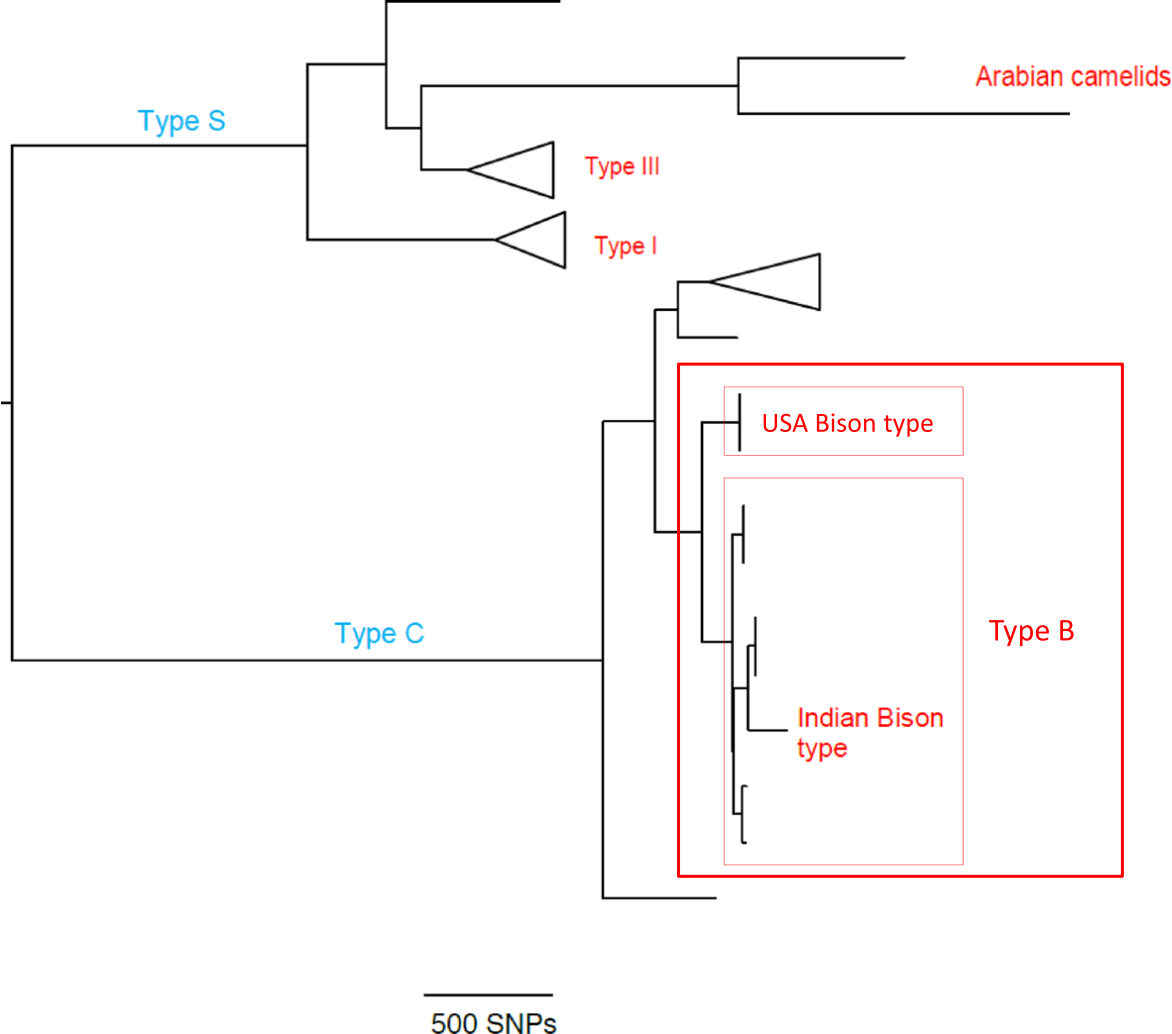
**The phylogeny of** ***Map*** **strain types.** Maximum likelihood tree of *Map* strains based on whole genome SNPs. Next generation sequencing data from unpublished and published strains were mapped against *Map K-10* reference genome, and SNPs called based on previously described filters [98]. The tree was built using RaXML vs 7.8.6 [99] with 100 bootstrap replicates. Branches were collapsed (indicated by triangles) and visualised using FigTree [100].

Over the past two decades various strain types of *Map* have been differentiated using different molecular techniques. As a result a complex nomenclature for *Map* strains has evolved and the phylogenetic relationships between these strain types has been clarified only recently by whole genome sequencing (J Bryant, K Stevenson, unpublished observations; Figure 1). There are two major groups of strains known as “Sheep-type” or “Type S” and “Cattle-type” or “Type C” originally named after the host species from which they were first isolated [11]. The Type C group is synonymous with the Type II strains as defined by pulsed-field gel electrophoresis and described by Stevenson et al. [12]. A third group of strains termed “intermediate” [11] or “Type III” [13–15] was originally thought to be intermediate between Type S and Type C strains but whole genome sequencing has confirmed that it is actually a subtype of Type S strains (J Bryant, K Stevenson, unpublished observations; Figure 1). Similarly, the Type I pigmented ovine isolates from the UK described by Stevenson et al. [12] and the recently sequenced camelid isolates [4] also comprise sub-lineages of Type S (Figure 1).

Another group of strains designated “Bison” or “B-type” has been described in the literature [16,17]. These strains were first differentiated on the basis of the number of copies with a C or a T at base pair 223 in the insertion sequence IS*1311* [16]. All copies of IS*1311* in B- Type strains have a T at position 223 whereas the Type S strains all have a C and the Type C strains have one or more copies with a C or a T at this position. Further analyses of B-type isolates revealed that the B-type isolates from animals in India were different from those isolated from US bison and these became known as “Indian Bison type” [18]. More recently, Indian Bison Type strains have been identified by a TG deletion at base pair positions 64 and 65 of IS*1311* at locus 2 [19] and also by a number of single nucleotide polymorphisms (SNPs) identified by whole genome sequencing (C Leão, unpublished observations). The Indian Bison type strains are a sub-lineage of the Type C strains [20] (Figure 1).

Genome sequencing of human *Map* isolates from patients with inflammatory bowel disease has shown that these do not comprise a distinct strain type and cluster with Type C cattle isolates following phylogenetic analysis [21,22, J Bryant, K Stevenson, unpublished observations].

Type S and Type C strains can be differentiated by most typing systems but distinguishing between isolates within these strain types is more difficult and requires the use of more than one typing method and for PCR-based systems the detection of multiple loci [9,10]. Whole genome sequencing provides the ultimate resolution and has revealed genetic diversity among closely related *Map* isolates [21,22]. Throughout this review the two major strain types will be referred to as Type S and Type C and the Type I and III classification for Type S subtypes.

## 3. Source of genetic variability

Microbial genomes are plastic changing over time as a result of spontaneous mutations and in response to changing selective pressures within the microenvironment. Spontaneous mutations arise due to the chemical instability of the purine and pyrimidine bases and errors during DNA replication. Natural exposure of an organism to certain environmental factors such as UV light also can induce mutations.

Genetic polymorphisms in *Map* strains have been determined by microarray comparisons and in silico analyses of whole genome sequence data. The first *Map* strain to be sequenced was a Type C strain, *Map K-10* in 2005 [23]. Subsequent optical mapping revealed that the sequence contained an inversion due to mis-assembly [24] and the corrected sequence was published in 2010 [25]. Genome sequence data for *M.avium hominissuis* (*Mah*) strain 104 (*Mah 104*) is available in the NCBI Genbank (NC_008595). A draft sequence of a Type S strain (*Map S397*) was compiled following genome sequencing and optical mapping and published in 2012 [26]. These provide the reference genomes for sequence and genome comparisons and all variations are described relative to these.

### 3.1 Large sequence polymorphisms

Whole genome comparisons have identified large sequence polymorphisms (LSPs) including insertions, deletions, inversions, translocations and duplications, all of which contribute to the unique genotypic composition of *Map* isolates. Large-scale DNA rearrangements can disrupt the integrity of a large number of coding and regulatory genes affecting their function, which can result in major phenotypic consequences. Large-scale insertions can also result in the acquisition of new genetic material such as virulence and antimicrobial resistance genes that confer a selective advantage and contribute to the fitness of a pathogen in specific microenvironments. Horizontal gene transfer has been detected in mycobacteria [27] and a recent study by Wang et al. [28] has shown that the genetic elements required for in vivo survival of *Map* comprise a mixture of *Map*-specific genes acquired via horizontal gene transfer and conserved mycobacterial virulence genes. A number of LSPs have been identified between Type S and Type C strains. However, the genome is remarkably homogeneous between isolates within each of these lineages [21,22,26].

#### 3.1.1 Genomic insertions

Comparison of the whole genome sequences of *Map S397* (Type S) and *Map K-10* (Type C) reveals ten LSPs containing four or more open reading frames (ORFs) present in the Type S strain and *Mah* but absent from the Type C strain [26]. Four of these were identified previously by genomic hybridisation and microarray analyses [29–32]. The largest of these is a 9 kb fragment comprising 13 ORFs, which encodes a number of proteins of particular interest that could have phenotypic consequences including a TetR transcriptional regulator, PPE proteins, HspR protein, PapA2 protein, ABC-2 type transporter and IS*1311*. The polymorphism also truncates MAP2178 involved in mycobactin synthesis. Another LSP insertion of particular note contains 14 ORFs predicted to encode proteins involved in the biosynthesis of glycopeptidolipids, which promote macrophage activation in a TLR2 and MyD88-dependent manner [26,31,33]. Overall a total of 70 ORFs are present in *Map S397* but absent from *Map K-10* and of these 57 are present in *Mah* 104 and 13 unique to *Map S397* [26]. As more *Map* Type S strains are fully sequenced, it will be possible to confirm whether these ORFs are exclusive to all Type S strains.

#### 3.1.2 Genomic deletions

Whole genome comparisons between *Map S397* and *Map K-10* also reveal 32 ORFs present in *Map K-10* but absent from the genome of *Map S397* [26]. These included the previously described deleted regions MAP1484c to MAP1491 [31,32,34] and MAP1728c to MAP1744 [30–32,35]. Additionally, Castellanos et al. [32] reported the absence of MAP3584 from Type III strains compared with Type I strains. Other heterogenic loci identified in Type S strains include MAP1433c to MAP1438c [26,32], also known as VA15 [31] and MAP2325 [26,32,34,36]. Although discrepancies between microarray and genome sequence data cannot be ruled out, these differences may represent intra strain variations due to geographical differences or alternatively may have been derived through the process of host adaptation and reflect true functional differences between strains.

#### 3.1.3 Genomic inversions

Genome sequencing and optical mapping of *Map S397* revealed 14 inversions compared with the corrected genome sequence of *Map K-10* [25,26]. These inversions vary in size from 22 to 1,174 kb and span a total of 2.4 Mb of the S397 genome. Seven of the inversions are larger than 22 kb and 13 have at least one IS element on the flanking regions. Inversions result in a change in genome synteny (gene order) and in mycobacteria appear to be mediated by insertion sequences. Mycobacteria may utilise inversions to change the expression of certain genes for the benefit of the bacterium during infection. It is possible that the large gene inversions could be responsible for antigenic variations between strain types, although this has to be verified at the transcriptomic and proteomic levels.

#### 3.1.4 Genomic duplications

Two large tandem duplications designated variable Genomic Island −17 (vGI-17) and vGI-18 have been identified in human *Map* isolates by Wynne et al. [21]. These duplications span 63 and 109 ORFs, respectively and both are flanked by an IS*4* element suggesting that transposon mediated recombination may be responsible for the heterogeneity of duplication between isolates. PCR screening of over 30 additional *Map* isolates of different provenance confirmed that the duplications are carried by many isolates. However, the proportion of cells containing vGI-17 varies between isolates leading the authors to hypothesise that the duplications are transient genomic arrangements that may confer a selective advantage for enhanced infection or persistence in a host.

### 3.2 Insertion sequences

Insertion sequences have proved to be important for distinguishing between mycobacterial species and subspecies. The *Map K-10* strain contains 19 different IS elements with 58 total copies including IS*1311* and the *Map*-specific elements IS*900*, IS*Mav2*, IS_*MAP02*, IS_*MAP04* and homologues of REP13E12. These IS elements are present in *Map S397* in the same copy number as *Map K-10* [26] and currently there is no evidence for IS elements unique to either Type S or Type C strains. However, copy number of IS*900* varies between Type C strains [37] and this has been exploited in some typing techniques such as restriction fragment length polymorphism coupled with hybridisation to IS*900*. Insertion sequences may insert into regulatory or coding regions of the genome and disrupt gene function or into intergenic regions causing minimal disruption. Additionally, they can effect DNA rearrangements by transposon mediated recombination.

### 3.3 Repeat sequences

Repeat sequences are an important source of variation in bacterial genomes and are exploited by various typing methods for strain differentiation and molecular diagnosis. Mycobacterial Interspersed Repetitive Units (MIRUs) are repeat sequences present in the intergenic regions dispersed throughout mycobacterial genomes and are distinguished from other repeat sequences in that they do not contain dyad symmetries and comprise small ORFs whose extremities overlap those of the contiguous ORFs and are orientated in the same direction as these ORFs [38]. Variable Number Tandem Repeats (VNTRs) are tandem repeats of 15–100 bp dispersed at multiple loci in the genome. Short-sequence repeats (SSRs) consist of simple tracts of 2–5 bp tandem repeats. The total number of different repeat loci and the copy number of each repeat can vary between *Map* isolates. MIRU-VNTRs and SSRs have been used extensively for typing *Map* isolates and can distinguish both between Type S and Type C and within Type C strains to some extent [9,39,40].

### 3.4 Single nucleotide polymorphisms

Single nucleotide polymorphisms (SNPs) are the substitution of one nucleotide with another or an insertion or deletion of a single nucleotide. Single nucleotide substitutions in protein coding sequences can be synonymous where the substitution does not result in an amino acid change in the protein or nonsynonymous where the substitution results in an amino acid change that may have an impact on the function of the encoded protein. SNPs involving insertions or deletions in protein coding sequences result in frameshifts that lead to significant alterations in the encoding protein and the translation of downstream genes. SNPs in non-protein coding DNA can also have functional consequences if, for example, they affect a regulatory element. Also, SNPs in non-translated regions or synonymous SNPs may impact function by inducing alterations in RNA structure that influence RNA stability and/or small RNA-based post transcriptional regulation. Not all SNPs will result in phenotypic changes that affect epidemiologic or pathogenic traits but they are an important source of genetic variability. SNPs provide the greatest variation within the genome and are often thought of as the raw material of evolution. More than 3000 SNPs differ between Type S and Type C strains [26] but more importantly, SNPs provide intra-strain genetic variation. There are around 1000 SNPs between the camelid isolates and *Map S397* [4] and around 1000 SNPs between Type I and Type III strains. The high resolution afforded by genome sequencing can distinguish *Map* isolates within a strain type that cannot be differentiated by standard typing procedures. Epidemiologically related isolates can differ by just ten SNPs.

### 3.5 Genome stability and mutation rate

The fidelity of replication and the growth rate will affect the rate at which genetic variants can arise within a given pathogen species. Since *Map* replicates very slowly with a doubling time of 22–26 hours or more [41] and demonstrates low genomic diversity, the mutation rate is likely to be slow. A mutation rate of less than 0.3 SNPs per genome per year, which falls in the range reported for *M.tuberculosis* [42] has been estimated based on the analysis of more than 100 *Map* genomes using the Bayesian MCMC programme BEAST and different phylogenetic models (J Bryant, personal communication). The mutation rate and hence genetic variation is also influenced by selective pressures from the microenvironment such as the host immune system or presence of antibiotics. There have been few studies on genome stability although genetic changes occurring after in vitro passage of *Map* strains have been reported [11,43–45]. In contrast to in vitro passage, no changes were detected by IS*900*-RFLP analysis following in vivo passage [45,46]. However, further studies using genome sequencing with greater resolution will give a better estimate of genome stability. With respect to the short term stability of genotyping target repeat sequences and their suitability for epidemiological studies, MIRU-VNTR loci tested thus far appear to be stable enough for use in *Map* epidemiology, but the SSR locus 2 has been reported to be too unstable for this purpose [45].

## 4. Genetic variability and growth characteristics

The existence of at least two *Map* strain types differing in their growth characteristics, colony pigmentation and disease presentation was suspected as early as the 1930s but not confirmed until appropriate media could be found to support growth of both strain types [47,48]. We now know these strain types to be Type S and Type C. Generally, Type C strains are relatively easy to isolate from clinical samples and grow more quickly than Type S strains producing visible colonies in 4–6 weeks depending on the initial inoculum and the medium used. Type S strains are more difficult to isolate, taking 16 to 52 weeks to produce detectable growth. Both strain types grow on media based on Middlebrook 7H9, 7H10 or 7H11. Middlebrook 7H11 supplemented with mycobactin J is probably the best solid medium for the primary isolation of *Map* isolates from small ruminants which may be infected by both strain types [49]. With the withdrawal of Bactec12B medium, Whittington et al. [50] have developed a new liquid culture medium (M7H9C) comprising a Middlebrook 7H9 base supplemented with casitone, albumin, dextrose, catalase, egg yolk, mycobactin J and a cocktail of antibiotics that is suitable for the primary cultivation of Type S and Type C *Map*. Mycobactin J is usually added to all types of culture media, although some researchers have reported growth on 7H11 without mycobactin [51,52]. Type S strains grow poorly if at all on Herrold’s Egg Yolk Medium (HEYM), in contrast to Type C strains [48,49,52–55] and the addition of sodium pyruvate to the culture media can be inhibitory to Type S strains [49,52,56].

A recent study by Abendaño et al. [57] reported variable growth rates of low passage Type S and B-type isolates obtained from cattle, goat and wild animals but very similar growth rates for ovine isolates of Types S, C and B, although generally the Type S strains grew more slowly. The authors hypothesise that the isolates from sheep may have adapted to growth in this particular host by modifying the expression of some genes that might subsequently affect the strains’ ability to grow in liquid media. This may be reflected in the genotypes of these strains but whole genome sequencing and further investigations are needed to confirm this hypothesis.

Culture environments certainly influence *Map* genome diversity when strains are propagated in vitro over long periods. Long term systematic subculture of vaccine strains has influenced *Map* genome diversity resulting in large tandem genomic duplications, deletions and transposable element activity [44]. The vaccine strain 316F has been propagated on different media in different laboratories. A large deletion (vGI-19) is uniquely present in a 316F strain maintained on Dubos medium with added pyruvate and it is possible that this medium has been selective in that the deleted region includes homologues of glyoxylate enzymes associated with pyruvate metabolism. Similarly, another 316F strain maintained exclusively on potato starch medium has a unique large tandem duplication (vGI-22) containing extra copies of 14 ORFs including genes required for cell wall and fatty acid biosynthesis.

Another study has reported specific epigenetic distinctions between *Map* isolates from tissue versus faeces [58]. Amplified fragment length polymorphism (AFLP) analyses of *Map* isolates revealed genetic regions unique to tissue-associated isolates which showed no differences in terms of DNA sequence between *Map K-10* or faecal isolates. Further investigation demonstrated undigested AFLP restriction sites in the tissue-associated regions prompting the authors to speculate that methylation of these sites might be responsible. The discovery of a consensus sequence for possible methyltransferase recognition upstream of the undigested restriction site in the tissue-associated *Map* isolates would appear to support this hypothesis.

Colony phenotype does not appear to differ much between *Map* strain types, although the size and colour of colonies varies according to the media on which the strains are grown [49,52]. The exception to this are the distinctive pigmented strains that have been isolated from sheep, which produce a yellow or orange pigment that is stable during both in vitro and in vivo passage. The gut mucosa of sheep clinically infected with these strains is typically a brilliant yellow colour caused by the large number of pigmented bacteria present. Initially, pigmentation was thought to be a feature of Type I strains but a few pigmented strains have since been typed as Type III [40] and non-pigmented Type I strains identified (K Stevenson, unpublished results). No genetic polymorphisms or the presence or absence of any single gene which could be exclusively associated with the pigmented strains have been identified and the underlying reason for pigmentation in these strains remains unknown (J Bryant, personal communication).

*Map*, like other bacteria, emits volatile organic compounds (VOCs) during growth and a study by Trefz et al. [59] showed that 34 such compounds could be identified as biomarkers for *Map* growing on HEYM and that 2-ethylfuran, 2-methylfuran, 3-methylfuran, 2-pentylfuran, ethyl acetate, 1-methyl-1-H-pyrrole and dimethyldisulfide correlated with density of bacterial growth. Only a limited number of *Map* strains were investigated; a laboratory adapted Type C reference strain (ATCC 19689), one Type S (sub-type III) and 3 Type C field strains of different genotypes. However, the *Map* strains could be differentiated on the basis of their VOC emission patterns indicating that *Map* strains vary with respect to growth and metabolic activity. The Type C reference strain and Type S strain could be distinguished from the Type C field strains but the emissions from the Type C field strains were not markedly different.

## 5. Genetic variability and epidemiologic traits

*Map* strain differences in epidemiologic traits such as preferred host species and transmission factors have been suggested, but the results of past epidemiological studies have to be reviewed with caution since they did not always employ media that would support the growth of all strain types or sufficiently discriminative typing techniques.

Type C *Map* strains show no host preference and can be isolated from a broad range of domesticated, captive and free-living wildlife species, including non-ruminants [1–3]. They are usually the predominant strain type isolated from cattle. The Bison subtypes similarly are not restricted to *Bison* species and have been isolated from many domestic and wildlife species and humans [18,60–62]. There is mounting evidence for interspecies transmission of Type C strains. The same strains have been found to infect wildlife species and domestic ruminants on the same property [63,64], between two species of farmed ruminants on the same farm [49,63], and between wildlife species on the same property [63]. High resolution genome sequencing will no doubt reveal many more.

Type S strains have been predominantly isolated from sheep and goats in the past and this has led to a perception that they have a preference for these host species. However, Type S strains have been shown to produce clinical disease following experimental infection of cattle [65] or deer [66]. There has been one report of the isolation of a pigmented *Map* strain from a cow [67] and this strain was also experimentally transmitted to sheep. Experimental infections utilise high doses of in vitro grown *Map* and may not accurately reflect the situation in the field. In terms of natural infections, Type S infections of cattle [68–70], farmed and wild deer [70,71] and Arabian camelids [4] have been reported. The most convincing evidence for interspecies transmission of Type S strains comes from the scenario following the importation of Karakul sheep from Germany to Iceland in 1938 [72]. The Icelandic strains were characterised retrospectively as Type S strains [69]. After their introduction in sheep they passed to the local cattle population and subsequently became endemic. The risk of natural transmission of Type S strains from sheep to cattle was believed to be low and to occur when susceptible animals were exposed to high doses [73]. However, a recent study by Verdugo et al. [70] suggests that interspecies transmission of Type S strains may not be such a rare event if there is close contact between different species at the farm level. This study reported that Type S strains are more frequent in New Zealand beef cattle than Type C and that the same S subtypes were present in beef cattle and sheep co-grazing on farms. Farming systems therefore have an impact on strain transmission with co-grazing two or more ruminant species increasing the risk of interspecies transmission. These studies [69,70, 73] do not differentiate between the different S subtypes I and III, which could differ in their ability for interspecies transmission.

The transmission of *Map* also depends on the ability of the bacterium to survive within the environment and there appears to have been only one study investigating environmental survival of different *Map* strains. A study by Eppleston et al. [74] reported the survival of a Type C *Map* strain was not affected by site but for a Type S strain the hazard of death was 2.3 times higher at arid zone sites compared with temperate sites in Australia. *Map* has the capacity to form spore-like morphotypes that are resilient to heat and could facilitate survival within the environment [75]. Information on the survival of the different *Map* strain types in soil, water, silage and manure is of significant importance for reducing transmission and requires further research.

The geographical distribution of *Map* strains has probably been influenced by many factors including animal movements, farming practices and strain virulence. Currently, whole genome sequencing has shown that there is little evidence for geographically distinct strains (J Bryant, K Stevenson unpublished observations). The pigmented Type I strains do appear to be geographically restricted to pockets within the UK and are rarely found elsewhere. Distinct Type C genotypes have been found for Canada [76] and the Azores, Portugal (C Leão, personal communication). However, the picture may change as more *Map* isolates are subjected to whole genome sequencing.

## 6. Genetic variability and virulence

There is a paucity of information on differences in the abilities of *Map* strains to cause disease due in part to the challenges of differentiating between strain types and assessing virulence both in the field and using animal models. Few data sets exist that combine information on *Map* genotype and the outcome or severity of disease in the field and virulence has mostly been determined for laboratory cultured strains using animal models. There appears to be differences between Type S and Type C strains with respect to their virulence in different host species. Experimental infection of deer with a Type C isolate established infection in 100% of the animals whereas the infection rate was 69% with the same dose of a Type S strain, suggesting that Type S strains may be less virulent for deer or alternatively deer may be more resistant to infection with this strain type [66]. A recent study assessed the influence of different *Map* strains on the pathogenesis of disease in sheep, through the evaluation of lesion development and peripheral immune responses [77]. The researchers found specific antibody and interferon-γ (IFN-γ) production was significantly higher in lambs infected with Type C strains whereas no consistent IFN-γ responses were measured in those infected with Type S strains. In Type S infected lambs the granulomatous lesions were focal and restricted to lymphoid tissue with no differences in lesion intensity over time. In contrast, in lambs infected with Type C strains the lesions were more severe at 150 days post inoculation while at 390 days post infection lesions had decreased in severity and were characterised by well demarcated granulomas with fibrosis, suggesting lesion regression. Differences in lesion type were observed between a Type C field strain and *Map K-10* at 150 days with the field strain producing diffuse lesions and the *Map K-10* producing less severe multifocal lesions, suggesting that the *Map K-10* may be less virulent possibly as a result of laboratory adaptation. No differences were observed between the two Type S (Type III) field strains in the study. The Type C lesions occurred in lymphoid tissue and in the lamina propria both associated with and not associated with lymphoid tissue. Infection with Type C strains was also characterised by the appearance of giant cells. These have been observed previously in leporine paratuberculosis [6] (also Type C strains), are common in natural bovine paratuberculosis and were reported in a previous infection study [78]. Since different Type C strains were used in these experimental infections, the presence of giant cells may be the consequence of *Map* strain type rather than host responses. Currently there are no published reports of similar experimental infections of calves to establish the effect of *Map* strain type on the pathogenesis of bovine paratuberculosis.

Differences in virulence between *Map* vaccine strains 316F, II and 2e (all Type C strains) with respect to a virulent *Map* Type C clinical isolate have been demonstrated using a mouse model of infection [44]. The vaccine strains were clearly attenuated with regard to their ability to survive and persist in mice as measured by a reduction in the numbers of bacteria recovered and numbers of leucocyte clusters containing acid fast bacteria in the livers. Strains 316F and 2e were also shown to be attenuated with respect to a virulent field strain in a calf model of infection [79]. Analyses of the genomes of these and other vaccine strains was accomplished using a pan *Map/Mah* microarray and multiple genomic variations specific for various vaccine strains and stocks were identified including deletions, tandem duplications, variable genomic islands and insertion sequence copy numbers [44]. These genomic variations give clues as to possible mechanisms of attenuation of these strains. Of particular interest is a deletion (vGI-20) spanning 34 ORFs, including ten *Map*-specific genes in vaccine strains 2e and II. This region includes part of the 38Kb pathogenicity island identified by Stratmann et al. [80] and the *Map*-specific genes could provide the basis for differentiating infected from vaccinated animals (DIVA). The deleted region contains genes potentially involved in virulence and pathogenesis including PapA2 (involved in cell adhesion), genes encoding the anti-host killing factors glyoxalase and catalase, genes involved in cell internalisation and fatty acid metabolism. Strain II additionally contained a large 41 ORF tandem duplication (vGI-21) which included duplication of genes involved in benzoate and lipid metabolic pathways and it is tempting to speculate that this duplication arose to compensate for the loss of the genes involved in lipid biosynthesis and carbon usage in the vGI-20 deletion. The vGI-20 deletion and the vGI-21 duplication were not present in 316F suggesting that attenuation in this strain is due to different genetic polymorphisms.

## 7. Genetic variability, infection and pathogenesis

Disease is the outcome of a battle between host and pathogen involving a complex interplay between host defence mechanisms and attempts of the pathogen to circumvent these defences. Key determinants of bacterial virulence are those that facilitate adhesion, invasion and colonisation of host cells. Evidence is emerging that there are differences in the host-pathogen interactions that could be attributed to different *Map* strain types. The first stage of *Map* infection is the invasion of the intestinal barrier via both microfold (M) cells of Peyer’s patches and differentiated epithelial cells [81]. During these early infection events bacterial adhesions can play a crucial role. The heparin binding haemagglutinin (HBHG) is one such adhesion located on the surface of the mycobacterium, which has been shown to mediate the binding of the bacterium to epithelial cells and fibroblasts [82]. The C-terminal regions of the *hbhA* gene differ between Type S and Type C *Map* strains mainly in deletions or differences in the lysine-rich repeats, which are important for the binding of HBHA to HS-GAG [83]. No differences could be found between the Type S subtypes I and III. The HBHA proteins from *Map* Type S and Type C strains were found to exhibit different binding activity with sulphated glycoconjugates, with Type S strains having the highest binding affinity correlating with a greater number of lysine-rich repeats [83]. Since HS-GAG structures differ according to both hosts and organs, it is possible that mycobacterial pathogens use heparin-binding domain variability to define their host preference or tropism for the intestine. Further investigations are required to further elucidate the role of this protein in pathogenesis of *Map* strain types.

Following invasion of the intestine, *Map* is then translocated to the submucosal macrophages where the bacterium encounters a hostile environment evolved to destroy and internalise pathogens. The survival of internalised *Map* depends upon its ability to inhibit phagosome acidification and phagolysosome fusion to avoid hydrolysis and oxidation. There is some evidence that different *Map* strains have different capacities for entry and survival in macrophages. Several studies have reported increased uptake and survival of *Map* Type C strains by bovine monocyte-derived macrophages (MDMs) compared with a Type S strain [84,85]. Furthermore, no effect of the origin of MDMs (Johne’s disease positive or control animals) on bacterial survival irrespective of *Map* strain type could be observed, suggesting that previous exposure of MDMs to *Map* had no impact on bacterial survival in vitro [85]. These studies employed very few strains and a more recent study investigating a larger panel of genotypically distinct strains from six different host species concluded that survival of *Map* isolates in bovine macrophages is associated with the specific host from which the isolates were initially isolated rather than genotype [86]. This study also reported that *Map* growth was less variable in BoMac cells (a SV40-transformed bovine peritoneal macrophage cell line) than MDMs. The discrepancies between these studies could possibly be explained by the variable results obtained using MDMs, the limited number of strains employed by the earlier studies or the difference in methodology used for enumerating survival of *Map* in macrophage cells. The importance of this methodology has been recognised and is now being addressed [86,87]. Clearly more research is needed in terms of entry and survival of different genotypically distinct *Map* strains of both Type C and Type S in macrophages especially in vivo.

Studies have been undertaken to investigate the response of different *Map* strains to the macrophage environment. Zhu et al. [88] used selective capture of transcribed sequences to investigate the responses of the same strain types used by Janagama et al. [84] and Gollnick et al. [85] within MDMs. Despite variations in the genes identified, in general the different *Map* strains responded in a similar fashion to the macrophage environment upregulating genes in cell wall biosynthesis and pathways that combat oxidative stress, metabolic and nutritional starvation and cell survival. Transcription of four genes was upregulated exclusively in Type C strains; MAP1728 (encoding YfnB, a predicted hydrolase), MAP1738 (MmpL5), MAP1729c and MAP1730 (hypothetical proteins) suggesting that these may be strain specific responses for survival within macrophages. These genes are located within one of the deleted genomic regions characteristic of Type S strains. Protein expression profiles of one Type S and one Type C strains under oxidative and nitrosative stress and the stressors of temperature flux, hypoxia, nutrient starvation mimicking the environment of macrophages have also been investigated [89–91]. Some proteins were found to be differentially regulated between *Map* C and S Types; 10 in response to oxidative stress [91], nine to nitrosative stress [91], 27 to temperature flux [90], 21 to hypoxia [89] and 26 to starvation [89]. Seven of these proteins were differentially regulated in Type S and Type C strains in response to several stressors: DesA2 to oxidative and thermal stress; AhpC to oxidative, nitrosative stress and starvation; AhpD to hypoxia, oxidative and nitrosative stress; Ppa to oxidative, nitrosative and thermal stress, hypoxia and starvation; FabG to nitrosative, thermal stress and starvation; hypothetical protein MAP2411 to oxidative stress and starvation and hypothetical protein MAP1885c to nitrosative stress and starvation. In terms of strain specificity, AhpD and FabG were only identified in the Type C strain under stress, suggesting that the regulation of these proteins is a general response to stress by Type C strains. Nutrient starvation inhibited the growth of both strain types but was lethal for the Type S strain after 12 weeks [89].

Another stressor encountered by *Map* infecting macrophages is the initial low availability of iron. Iron is essential for growth and a key cofactor in many enzymatic pathways including those involved in energy production and nucleic acid synthesis and is part of the active centre of stress-resistance proteins such as superoxide dismutase. Although *Map* requires exogenous mycobactin for in vitro growth, this may not necessarily be the case in vivo since the mycobactin synthesis genes (*mbt*) are upregulated inside bovine MDMs [88]. In addition, the concentration of iron within macrophages increases between one and 24 h following infection by pathogenic mycobacteria, suggesting that these bacteria possess mechanisms to acquire and concentrate iron in the phagosome [92]. Evidence is accumulating to suggest that the Type S and Type C *Map* strains differ in their iron regulatory mechanisms. Both *Map* strain types possess the iron-dependent global regulator IdeR, which binds to a 19 bp consensus sequence (the “iron box”) and regulates a repertoire of genes involved in iron acquisition (*mbt*) and storage ( bacterioferritin *bfr*A) in response to iron concentration [93]. Both the Type C and Type S IdeR repress transcription of the *mbt* genes involved in iron acquisition at high iron concentrations and relieve repression at low iron concentrations but polymorphisms within the promoter of *bfr*A in Type S compared with Type C affect gene expression resulting in defective iron storage in this strain type [93]. Janagama et al. [94] investigated the iron-sparing response of *Map* strain types by transcriptional and proteomic profiling under iron-deplete and –replete conditions. Under iron-deplete conditions, there was upregulation of siderophore synthesis and transport genes (*mbt*, *esx-3*, *irtA* and *irtB*) in both *Map* strain types but differential expression of aconitase, succinate dehydrogenase and superoxide dismutase, which were downregulated in Type C and upregulated in Type S. Under iron-replete conditions, there was an upregulation of *bfr*, Mycobacterial heme degrader, ribosomal proteins and the Antigen 85 complex in Type C strains but not in Type S strains. These results suggest that Type C strains have an efficient iron-sparing response in contrast to Type S strains, which potentially could give them a survival advantage. Interestingly, Type C strains also exclusively upregulated MAP2325 under iron-deplete conditions, which may also confer a survival advantage. This gene is an ortholog of the enhanced intracellular survival gene (*eis*) described in *Mycobacterium tuberculosis* (*Mtb*) that enhances survival in macrophages.

In addition to killing intracellular pathogens, macrophages are key players in antigen processing and presentation and orchestrating host inflammatory and immune processes. Different *Map* strain types appear to have varying influences on these host responses. Studies by Janagama et al. [84] and Borrmann et al. [95] investigated cytokine responses to different *Map* strain types in bovine MDMs and the human monocyte cell line THP-1, respectively by RT-PCR. Both studies reported that the Type S strain produced less IL-10 and more TNF-α than the Type C strains but obtained different results with respect to IL-1β, which could reflect further strain or host differences. The observed effects in THP-1 cells were also influenced by challenge dose and infection time [95]. Motiwala et al. [96] performed a genome-wide transcriptional analysis of THP-1 cells exposed to different *Map* strains and found that Type C strains induced anti-inflammatory and anti-apoptotic pathways in the cells without causing significant alterations in pro-inflammatory genes, which would favour bacterial survival and persistence. Conversely, Type S strains significantly up-regulated pro-inflammatory genes related to IL-6, T-cell receptor, B-cell receptor and death receptor signalling within THP-1 cells similar to *Mycobacterium avium* subsp. *avium*. Additional genotypically distinct isolates from cattle, human, bison and sheep were analysed by quantitative RT-PCR for seven differentially expressed genes for consistency. The trends were the same but there were differences in the relative amounts of the transcripts, suggesting more subtle intra-strain type differences.

Toll-like receptors (TLRs) play a critical role in the innate immune response and many other cellular processes important to mycobacterial pathogenesis including phagosomal maturation. Activation of TLR9 initiates responses crucial for defence against mycobacterial infection, whereas activation of TLR2 induces responses that suppress immune defence against mycobacteria. Thirunavukkarasu et al. [97] reported significant up-regulation of TLR2 in the peripheral blood cells of sheep experimentally infected with a Type S *Map* strain but in cattle experimentally infected with a Type C strain TLR2 was significantly down-regulated. At later stages of infection, TLR4 was significantly up-regulated in the Type S exposed sheep but no significant differences observed in the Type C exposed cattle. No differences in TLR9 expression were observed in either infected sheep or cattle. While these results may suggest *Map* strain related differences, the differences in expression could equally be due to the different host responses to infection.

## 8. Conclusions

It is clear that the *Map* genome is plastic and that genetic heterogeneity and phenotypic differences exist between *Map* strains. The number and scale of genomic polymorphisms are greater between Type S and Type C strains and this is reflected in the marked differences seen in phenotype affecting growth, virulence, infection, pathogenesis and epidemiological traits. Genetic diversity within each of these strain types is less easy to detect using conventional typing systems but genome sequencing has revealed genetic polymorphisms and SNP-based technologies will open the door to more discriminative strain differentiation. Currently, little data is available on the characteristics of individual genotypically distinct *Map* isolates of both Type S and Type C with respect to virulence, persistence, dormancy and associated host and clinical information. Genome wide association studies that focus on *Map* isolates for which this data is available would improve the identification of genes likely to be involved in strain-specific phenotypes.

Whilst it is often difficult to determine if a phenotype is due to the infecting *Map* strain or the host, overall there is sufficient evidence to conclude that the *Map* strain types influence host-pathogen interactions and the outcome of disease. This can occur at all stages from invasion of epithelial cells, uptake and survival in macrophages to the formation of pathological lesions. The two major strain groups, Type S and C, appear to have adapted differently to their microenvironments in order to survive and persist in the host. Survival outside the host in different environments is also an important prerequisite for *Map* and a key factor for transmission. The literature reviewed here hints that possibly Type C strains may be better at this than Type S strains, which do not survive well in arid conditions or when subjected to starvation. There is a dearth of information concerning the abilities of the different *Map* strain types to form spore-like structures and their survival in soil, water, silage and manure.

The genetic variability of different *Map* strains and their influence on infection and pathogenesis has important implications for diagnosis and control of Johne’s disease. Since the *Map* strains are not host-specific, it is important to be able to detect and control infection by either strain type in a single species and in other susceptible species. Diagnostic tests either detect the pathogen or the host response to the pathogen. Detection of the *Map* bacillus is routinely accomplished by culture and/or PCR. It is therefore necessary to ensure that a medium capable of supporting the growth of both *Map* strain types is employed and that the primers selected for PCR target either common genes or sequences such as IS*900* or use a combination of strain-specific genes if strain differentiation is required. Similarly, vaccines for Johne’s disease need to be effective against infections caused by both *Map* strain types and the selection of vaccine candidates needs to be done with care. A thorough understanding of the mechanisms of immune evasion and pathogenesis of both *Map* strain types is required to develop an effective vaccine. Current vaccines include heat inactivated formulations of the vaccine strain 316F and one of the disadvantages of the vaccine is that it is not possible to differentiate between infected and vaccinated animals. The genetic polymorphisms in 316F could be exploited to develop a DIVA diagnostic test to circumvent this problem. Information on *Map* strain virulence determinants and the immune profiles induced by the different *Map* strains is important for the rational design of novel vaccines. A number of genetic polymorphisms have been identified in both strain types that could have consequences with regard to their virulence and pathogenesis. Targeted studies are now required to determine the functional impact of these natural polymorphisms between strains.
